# Acute retinal pigment epitheliitis using adaptive optics imaging: a case report

**DOI:** 10.1186/s12886-024-03768-0

**Published:** 2024-11-25

**Authors:** P.A.T. Heutinck, S. Wooning, K. Liman, M. Durand, L. Sanchez Brea, C.C.W. Klaver, V.J.M. Verhoeven, D. Andrade De Jesus, A.A.H.J. Thiadens

**Affiliations:** 1 Department of Ophthalmology, Erasmus MC, University Medical Center, Rotterdam, The Netherlands; 2 Eye Image Analysis Group Rotterdam, Department of Radiology & Nuclear Medicine, Erasmus MC, University Medical Center, Rotterdam, The Netherlands; 3 Leiden Institute of Advanced Computer Science, Leiden University, Leiden, The Netherlands; 4 Imagine eyes, Orsay, France; 5 Eye Image Analysis Group Rotterdam, Department of Ophthalmology, Erasmus MC, University Medical Center, Rotterdam, The Netherlands; 6 Eye Image Analysis Group Rotterdam, Rotterdam Ophthalmic Institute, Rotterdam Eye Hospital, Rotterdam, The Netherlands; 7 Department of Epidemiology, Erasmus MC, University Medical Center, Rotterdam, The Netherlands; 8 Department of Ophthalmology, Radboud University Medical Center, Nijmegen, The Netherlands; 9 Institute of Molecular and Clinical Ophthalmology, University of Basel, Basel, Switzerland; 10 Department of Clinical Genetics, Erasmus MC, University Medical Center, Rotterdam, The Netherlands

**Keywords:** Adaptive optics flood illumination ophthalmoscopy, Acute retinal pigment epitheliitis, Krill’s disease, Cone photoreceptors

## Abstract

**Background:**

Acute Retinal Pigment Epitheliitis (ARPE, Krill’s disease) is a rare inflammatory retinal disorder commonly affecting young adults. It often presents unilaterally with central vision disruption, and typically resolves with vision restoration within 6 to 12 weeks. The pathogenesis of ARPE remains a subject of ongoing debate. Adaptive Optics Flood Illumination Ophthalmoscopy (AO-FIO) imaging has emerged as a valuable tool capable of detecting early cone photoreceptor changes and recovery. This case study presents two patients with ARPE, with longitudinal follow-up using multimodal imaging, including optical coherence tomography (OCT) and AO-FIO.

**Case presentations:**

A 30-year-old male presented with sudden vision loss in both eyes. The best corrected visual acuity (BCVA) was 20/33 and 20/40 Snellen in the right and left eye, respectively. OCT showed interruption of the ellipsoid zone (EZ) band and outer nuclear layer (ONL) in both eyes; AO-FIO imaging revealed a foveal lesion and diminished parafoveal cone density in both eyes compared to two age-matched controls. After 6 months, BCVA was restored to 20/20, and OCT showed recovery of the ONL and EZ. On AO-FIO, the foveal lesion was still present and the parafoveal cone density increased but remained reduced even up to 15 months after onset when compared to the controls. The second patient, a 30-year-old woman, presented with a unilateral drop in vision to 20/63 Snellen. OCT showed discontinuation of the EZ and hyperreflectivity within the ONL and retinal pigment epithelium in the affected eye. The unaffected eye showed no abnormalities. After 3 months, the BCVA improved to 20/16 Snellen and OCT showed recovery of the EZ. AO-FIO was conducted 9 months after onset and revealed reduced parafoveal cone density in the affected and non-affected eye compared to the controls while OCT still showed recovery of all retinal layers.

**Conclusions:**

ARPE is a self-limiting disease with recovery of BCVA and OCT retinal layers within 6 months. However, our 2 cases showed that parafoveal cone density recovered during follow-up but did not reach levels observed in controls. AO-FIO is an imaging modality that enhances sensitivity in measurements and can therefore be used as a complementary tool for follow-up.

## Background

Acute Retinal Pigment Epitheliitis (ARPE, Krill’s disease) represents a rare, self-limiting inflammatory retinal disorder predominantly observed in young adults. Its initial documentation dates to 1972, credited to Alex E. Krill and August F. Deutman [1]. In approximately 75% of cases, ARPE manifests unilaterally, typically characterized by central vision disruption or central scotoma. Clinically, the macula shows discrete clusters of small grey spots at the retinal pigment epithelium (RPE) level, encircled by hypo-pigmented yellow haloes. Optical coherence tomography (OCT) typically reveals a hyperreflective lesion at the outer retinal layer (ONL) and an interruption of the ellipsoid zone band (EZ). Resolution of these lesions and restoration of best corrected visual acuity (BCVA) to normal levels occur usually within 6–12 weeks in approximately 80–90% of patients [2, 3].

The precise pathogenesis of ARPE remains debatable. Adaptive Optics Flood Illumination Ophthalmoscopy (AO-FIO) emerges as a promising tool enabling the visualization and assessment of cone parameters otherwise inaccessible through conventional imaging modalities [4]. Various studies have highlighted AO-FIO’s ability to detect early alterations in photoreceptor density preceding visible clinical manifestations or abnormalities discerned via alternative imaging techniques [5]. AO-FIO imaging holds potential in augmenting our understanding by investigating photoreceptor dynamics.

To elucidate its clinical utility, we integrated AO-FIO imaging (rtx1 camera - Imagine Eyes, Orsay, France) into our standard imaging protocols, thereby facilitating a comprehensive evaluation of ARPE progression within the retina. Herein, we present two cases of ARPE, incorporating longitudinal multimodal imaging: scanning laser ophthalmoscopy (SLO), fundus autofluorescence (FAF), OCT, fluorescent angiography-indocyanine green (FA-ICG), and AO-FIO.

## Adaptive optics

Both patients underwent evaluation at the ophthalmic outpatient clinic of the Erasmus MC, where AO-FIO imaging was performed using the rtx1 retinal camera. In the case of patient 1, AO-FIO imaging of both eyes was performed at the initial consultation and continued during each subsequent follow-up visit up to 15 months following the onset of symptoms. Conversely, for patient 2, AO-FIO imaging was conducted nine months after onset of symptoms. Additionally, two age-matched healthy controls were enlisted for comparative analysis. A standardized AO-FIO protocol was uniformly applied to both the ARPE patients and the healthy controls to ensure consistency and reliability in the assessment process.

**Fig. 1:**
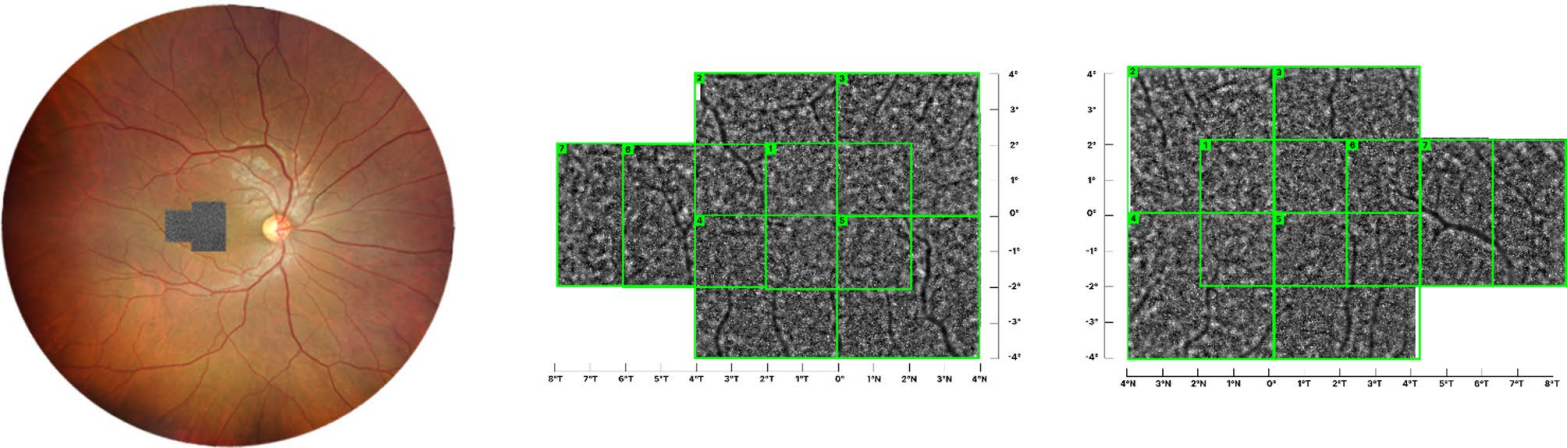
Seven images were captured per eye. The integration of these individual images into a single composite montage is depicted. Within the montage, the green squares symbolize individual AO images of 4° x 4°

Given that the ARPE lesions identified via OCT were situated in the fovea, AO-FIO imaging was conducted accordingly. The AO-FIO imaging protocol comprised seven images per eye, each covering a field of view measuring 4° x 4° (Fig. 1). Ensuring fixation alignment with the fovea, a single image was captured at coordinates 0°,0°. From the fovea (0°,0°), the protocol extended up to four degrees nasally and eight degrees temporally, as well as up to four degrees superiorly and inferiorly (Fig. 1). An image quality check was performed by the examiner during acquisition. All seven images were combined to create montage images for analysis of cone density using i2K retina AO (Dual Align, USA). To facilitate montage construction, an overlap between the images, up to a maximum of 2 degrees, was used (Fig. 1).

Parafoveal cone density measurements were done by using AO-FIO ConeDetect [6]. This tool is based on a deep learning–based model for detecting cone photoreceptor cells in AO-FIO. The deep learning model, based on the U–Net architecture, was trained on data from 625 AO-FIO manually annotated patches (128 × 128 pixels) and validated on data annotated by three different centers: Erasmus MC, The Netherlands, Ghent University Hospital, Belgium, and Lions Eye Institute, Australia. The training and validation data consisted of AO-FIO images from healthy eyes, all acquired using the rtx1 camera. Cone density maps were created for the montages of the APRE patients and the healthy controls to visualize the cone density measurements. Additionally, parafoveal cone density was measured at different eccentricity ranges (2°-3°, 3°-4°, 5°-6°, 6°-7°, 7°-8°) for comparison. The central 4° region (2° radius) was masked on all resulting montages, due to the limitation of the rtx1 camera in counting densely packed foveal cones. To assess the normal variability in cone density in a healthy individual, we used one year follow-up data from control 1.

## Case presentation

### Patient 1

A 30-year-old male patient presented with a sudden bilateral decline in BCVA, photophobia, color vision aberrations, and the emergence of a central shadow-like scotoma within his central visual field, all occurring over the course of one week. The patient had no relevant ocular or medical history. About three weeks before these symptoms began, the patient experienced an influenza episode. Four days before his presentation at our care facility, the patient began treatment with prednisone eye drops, as prescribed by the ophthalmologist by whom he was referred.

The BCVA measured 20/33 Snellen in the right eye (OD) and 20/40 Snellen in the left eye (OS). Slit lamp examination yielded unremarkable findings. Fundus examination revealed macular abnormalities. Considered diagnoses were ARPE, multiple evanescent white dot syndrome, punctate inner choroidopathy, or chorioretinitis.

Laboratory blood tests (including infectious disease and uveitis panels) were normal.

**Fig. 2:**
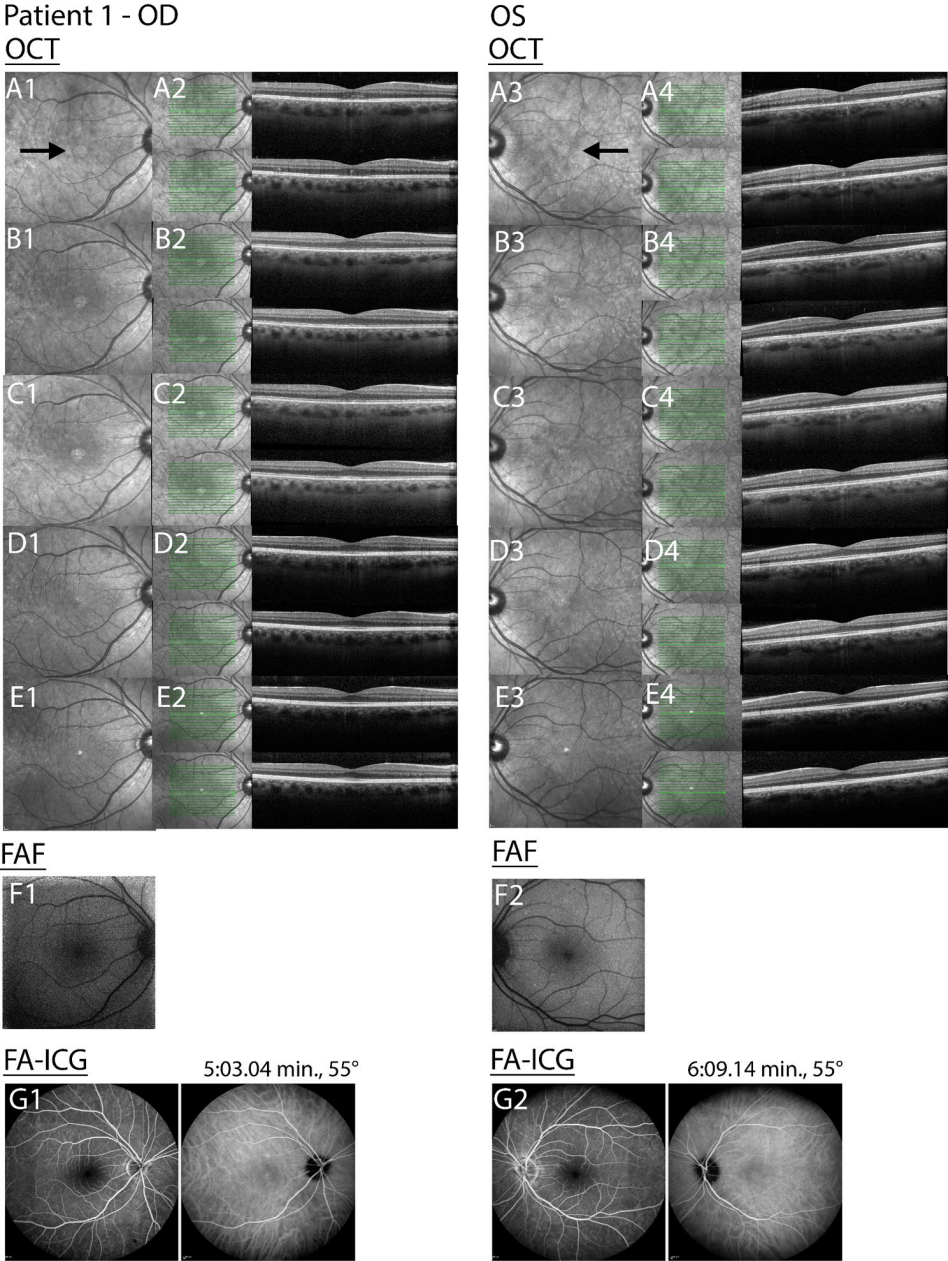
Disease progression patient 1. Time after onset of symptoms: A = baseline, B = 2 weeks, C = 1 month, D = 6 months, E = 15 months, F = 2 weeks, G = 1 month. The black arrows in image A1 and A4 point to the small hyperreflective dots. Right eye = OD; Left eye = OS; Optical coherence tomography = OCT; Fundus autofluorescence = FAF ; Fluorescent angiography-indocyanine green = FA-ICG; Min = minutes

**Fig. 3:**
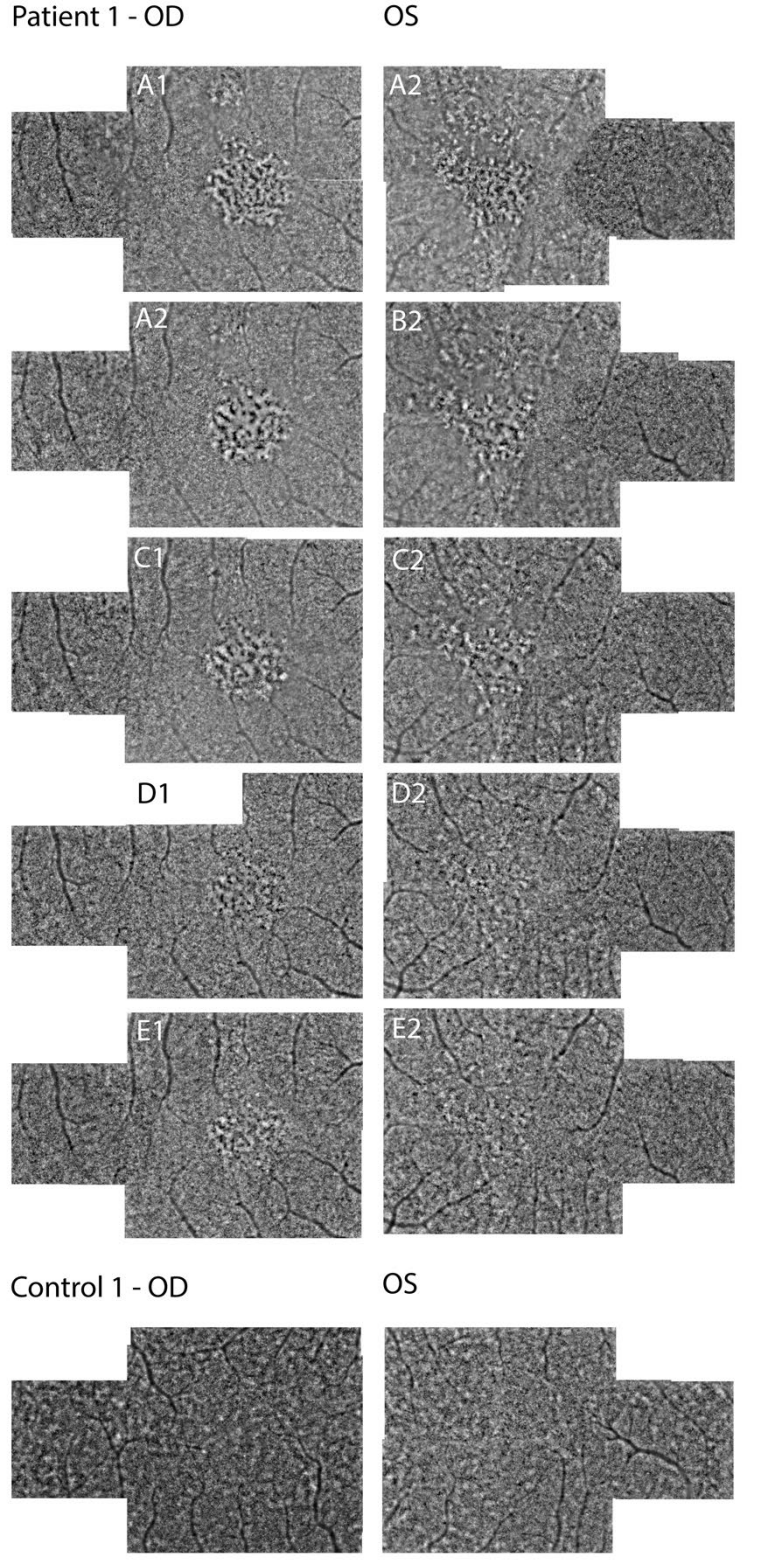
Adaptive optics montages of patient 1 and of control 1. Time after onset of symptoms: A = baseline, B = 2 weeks, C = 1 month, D = 6 months, and E = 15 months. Right eye = OD; Left eye = OS

**Fig. 4:**
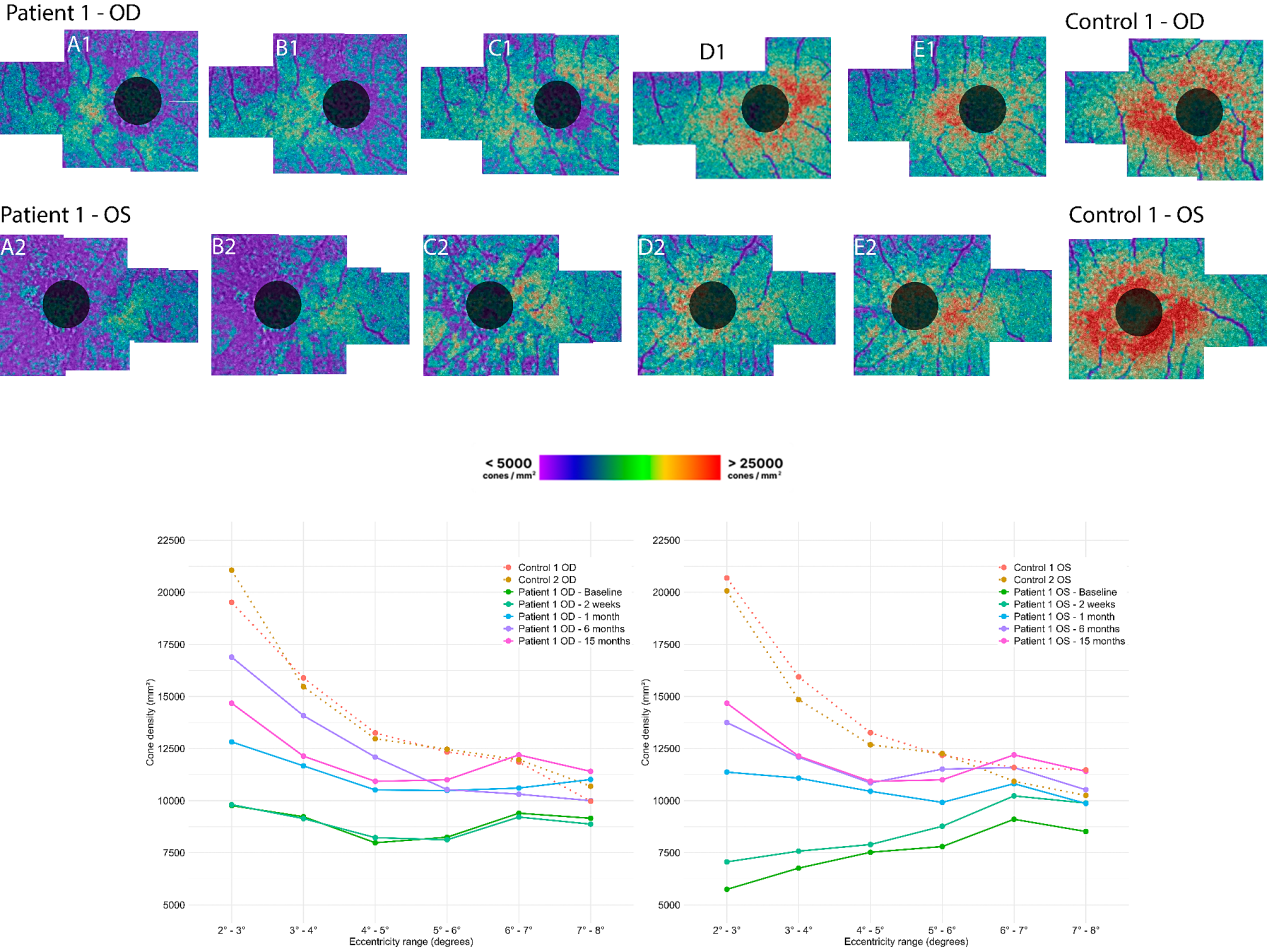
The upper section displays the cone density maps of patient 1 compared to control 1, with black circles representing the masked 4° central region. The lower section presents the cone density measurements of patient 1 (solid lines) at different eccentricity ranges compared to two age-matched controls (dotted lines). Time after onset of symptoms: A = baseline, B = 2 weeks, C = 1 month, D = 6 months, and E = 15 months. Right eye = OD; Left eye = OS

**Fig. 5:**
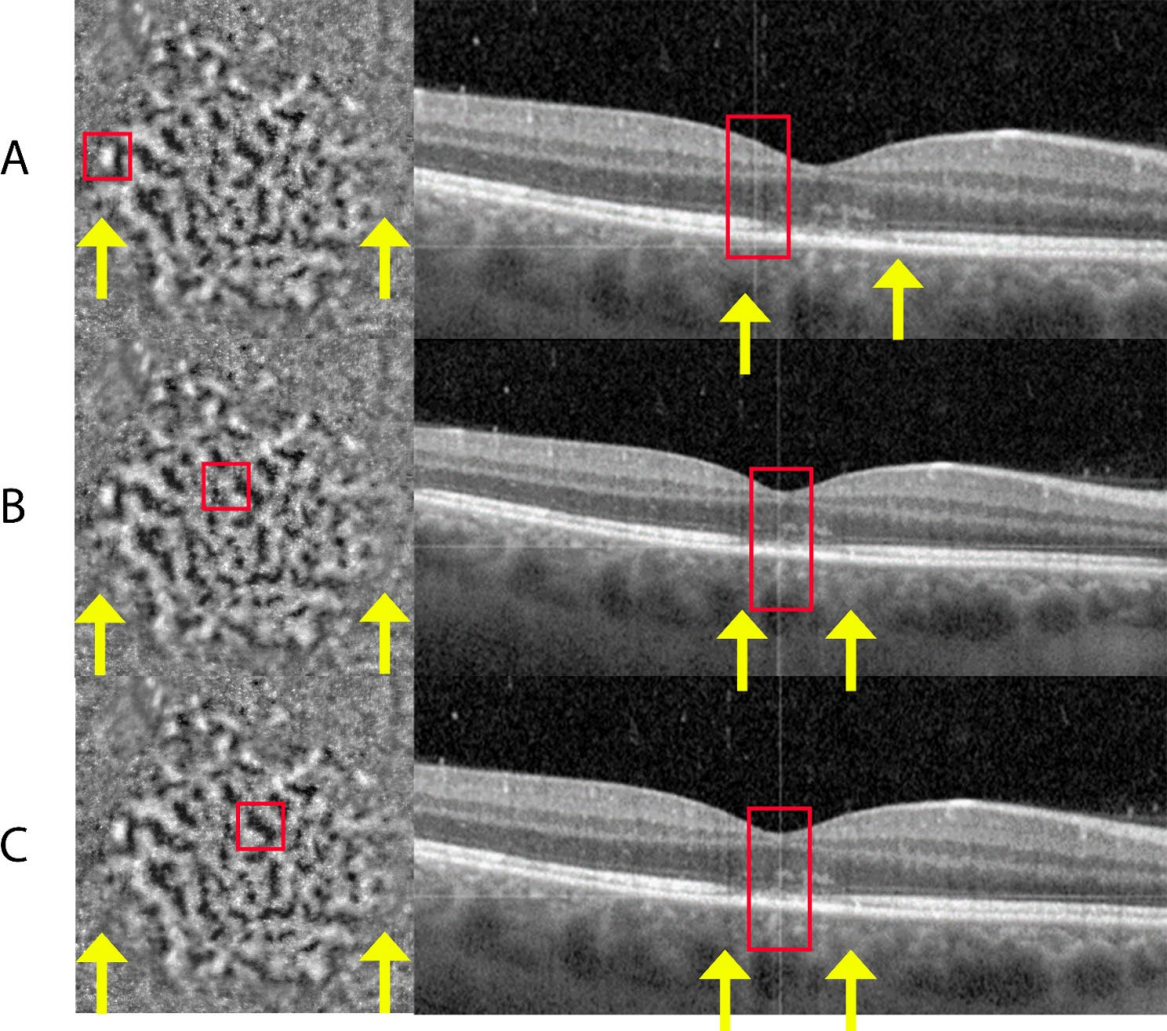
Visualizing the correlation between adaptive optics flood illumination ophthalmoscopy and optical coherence tomography findings. The lesion borders correspond to the retinal layer disruption on OCT (yellow arrows). **A**: The hyperreflective region on AO corresponds to ellipsoid zone (EZ) band interruption on OCT. **B**: The cone mosaic on AO corresponds to EZ disruption on OCT. **C**: The hyporeflective areas on AO correspond to irregularities in the outer nuclear layer (ONL) and interdigitation zone (IZ) on OCT

**Fig. 6:**
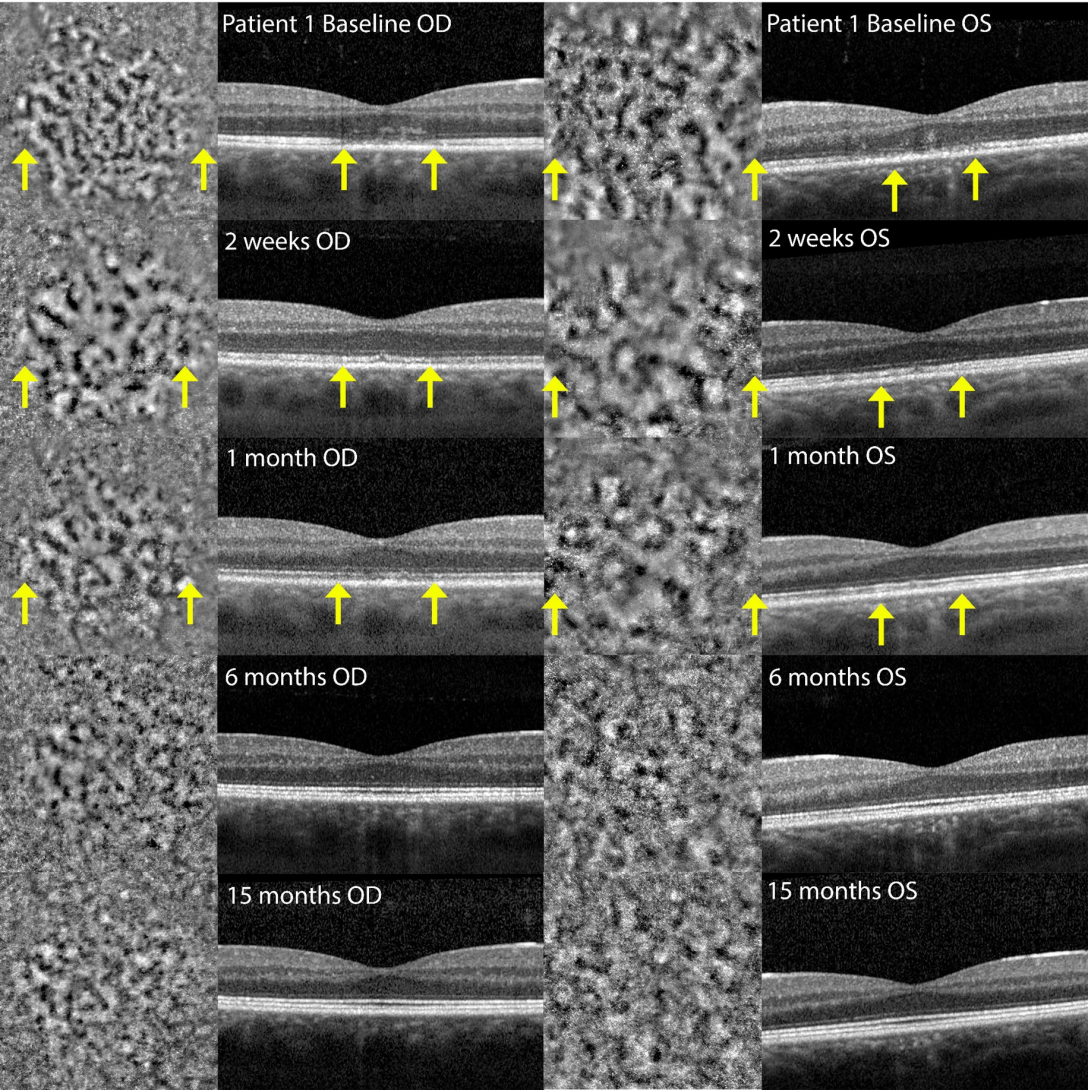
Follow-up of adaptive optics flood illumination ophthalmoscopy (AO-FIO) images focused on the foveal lesion in both eyes of patient 1. The lesion borders corresponded to the disruption of retinal layers on optical coherence tomography. After six months the retinal layers were restored; however, the lesion remained visible on AO-FIO. Right eye = OD; Left eye = OS

Infrared fundus imaging showed the presence of small hyperreflective dots in the fovea (Fig. 2: A1/A4). OCT revealed hyper-reflectivity in the ONL in both the fovea and parafoveal regions. Additionally, an irregular pattern of the RPE was observed in the fovea, along with disruption of the EZ and interdigitation zone (IZ) (Fig. 2: A2/A4). FA indicated no transmission or window defects while FA-ICG showed hypercyanence patch/halo like lesions (Fig. 2: G1/G2). FAF revealed hyper-auto fluorescence spots in the macula and near the vasculature (Fig. 2: F1/F2). AO-FIO imaging showed a foveal lesion with disruption of the cone photoreceptor mosaic in both eyes (Fig. 3A1/A2). Parafoveal cone density measurements were lower in all eccentricity ranges measured at baseline compared with age-matched controls (Fig. 4). The foveal lesions presented hypo- and hyperreflective spots, with granularity resembling abnormal cone mosaic pattern and were situated at the same locations as the disruption of layers observed on OCT (Figs. 5 and 6). When correlating these AO features with features observed on OCT through visual inspection, we noted irregularities in the ONL and disruption of the IZ at the sites corresponding to the hyporeflective areas on AO-FIO. Furthermore, at locations where hyperreflective spots appeared at the edge of the foveal lesion, interruption of the EZ band was evident on OCT. Additionally, within the lesion where disrupted cone mosaic-like pattern was discernible, disruption of the EZ band was also observed (Fig. 5).

Based on the phenotype observed in the ophthalmic examinations and multimodal imaging results, a diagnosis of ARPE was established. As ARPE is known as a self-limiting disease, no therapeutic interventions were recommended. The prednisone eye drops were stopped as they are not proven to be effective for this indication.

One month following the onset, the BCVA improved to 20/20 Snellen in both eyes. The shadow-like spot gradually disappeared. Improvement was observed in both eyes on OCT imaging, particularly in the outer segments and inner segment layer, with the resolution of irregularities in the RPE (Fig. 2C2/C4). After six months, the BCVA improved to 20/17 Snellen in both eyes, but the patient still noticed a difference in vision quality (color and resolution of the image). Fundus examination revealed very subtle RPE irregularities centrally in the macula in both eyes. OCT imaging demonstrated intact inner segment EZ and RPE in both eyes (Fig. 2D2/D4). AO-FIO imaging showed a lesion still visible in the fovea (Fig. 3D1/D2). Parafoveal cone density measurements recovered during follow-up, but did not reach density levels comparable to the age-matched controls (Fig. 4). After 15 months, the cone density measurements were comparable to those taken at 6 months (Fig. 4).

### Patient 2

**Fig. 7:**
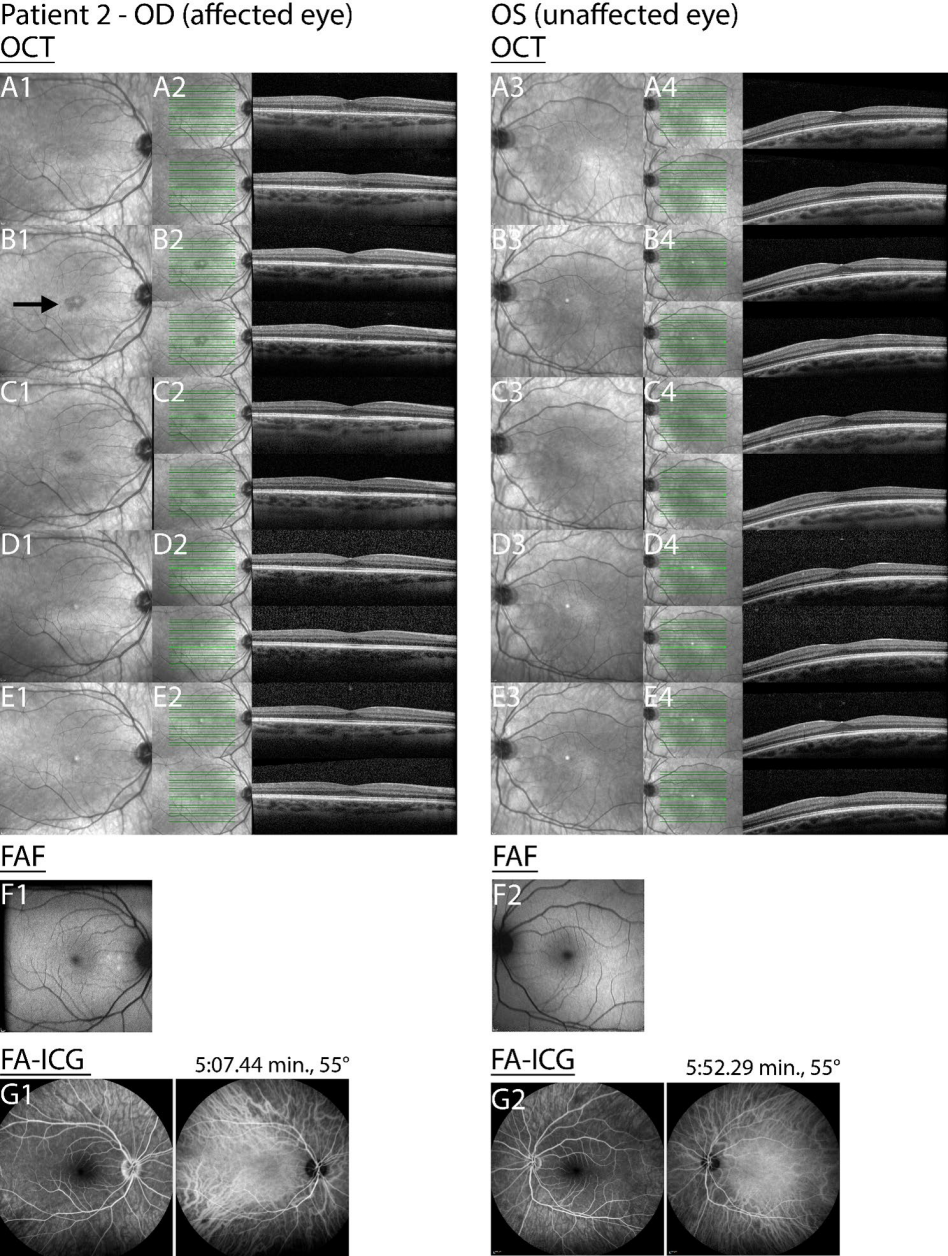
Disease progression of patient 2. Time after onset of symptoms, A = baseline, B = 2 weeks, C = 1 month, D = 6 months, E = 9 months, F = baseline, and G = 1 month. The black arrow in image B1 point to the lesion in the affected eye. Right eye = OD; Left eye = OS; Optical coherence tomography = OCT; Fundus autofluorescence = FAF ; Fluorescent angiography-indocyanine green = FA-ICG; Min = minutes

A 30-year-old woman, presented with a complaint of observing a grey spot accompanied by white dots in the central area of her OD over the past four days. She reported no complaints regarding her OS but mentioned feeling slightly unwell. She had no relevant ocular or medical history and was not undergoing any medication regimen. BCVA in the OD was 20/63, OS 20/22 Snellen visual acuity. Slit lamp examination revealed no abnormalities in both eyes. Infrared fundoscopy showed a hypopigmented lesion in OD two weeks post-onset of symptoms (Fig. 7B1). OS did not show abnormalities at baseline and two weeks post-onset of symptoms (Fig. 7A3 and B3).

FAF imaging of OD indicated three hypofluorescent spots (Fig. 7F1). OS did not show abnormalities (Fig. 7F2). OCT imaging of the OD showed a foveal discontinuation at the EZ and IZ, hyper reflectivity within the ONL and RPE (Fig. 7A2). OS did not show abnormalities (Fig. 7A4). FA-ICG revealed no signs of vasculitis or other abnormalities for both eyes (Fig. 7G1 and G2). A diagnosis of ARPE was established based on the phenotype observed in the ophthalmic examinations and multimodal imaging results. No therapeutic intervention was administered.

One month later, OCT imaging showed improvement of the RPE layer, with persistent disruption of the IZ, EZ and ONL in OD (Fig. 7C2). No abnormalities were observed in OS (Fig. 7C4). After three months the BCVA improved to 20/16 in OD, the BCVA in OS remained unchanged. OCT of OD showed recovery of EZ and IZ (Fig. 7D2).

**Fig. 8:**
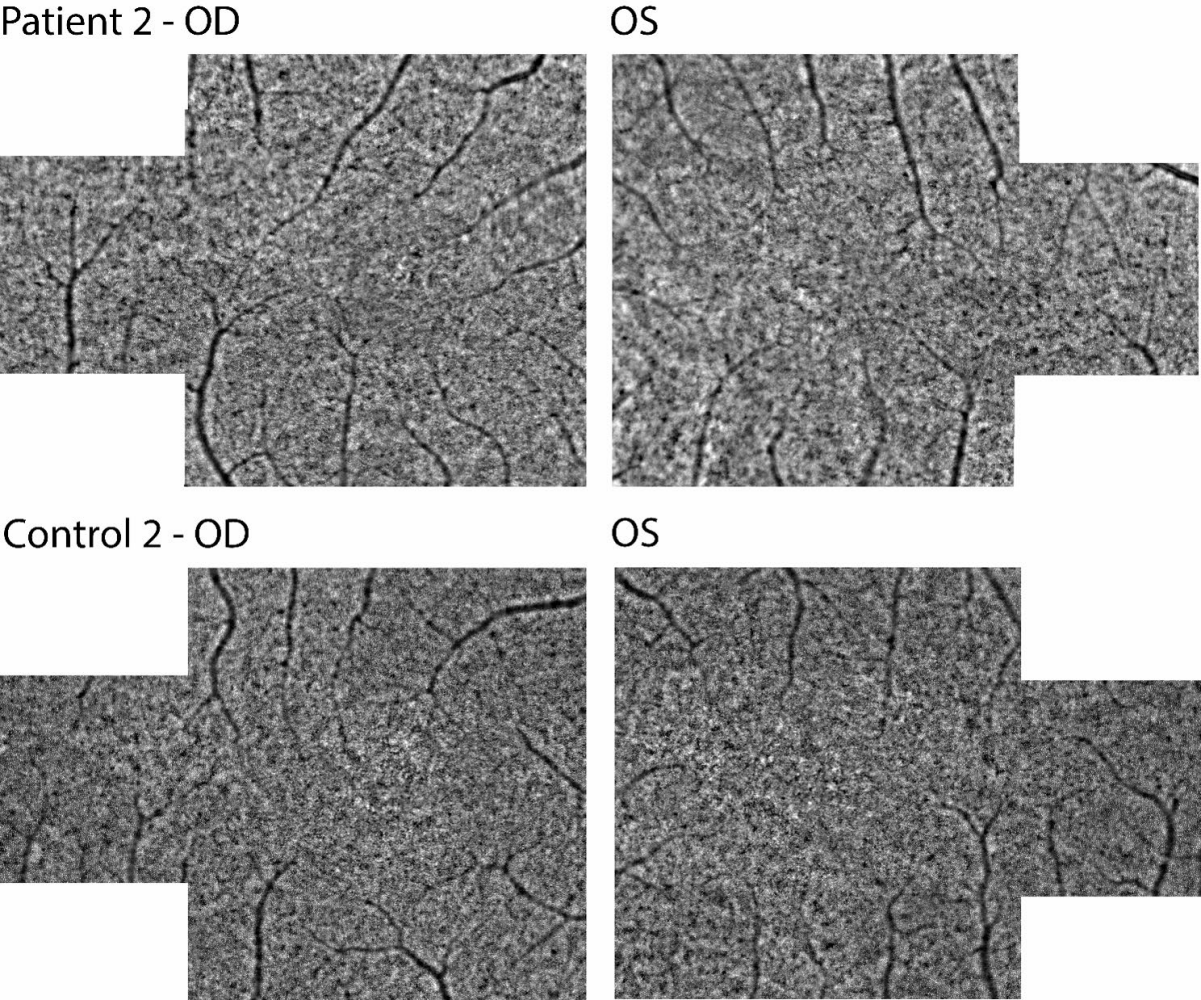
Adaptive optics montages of patient 2 nine months after onset of symptoms and of control 2. Right eye = OD; Left eye = OS

**Fig. 9:**
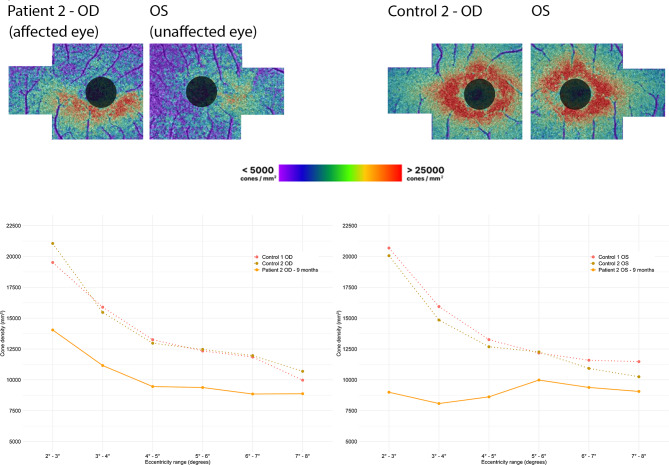
The upper section displays the cone density maps of patient 2 nine months after onset of symptoms compared to control 2, with black circles representing the masked 4° central region. The lower section presents the cone density measurements of patient 2 (solid lines) at different eccentricity ranges compared to two age matched controls (dotted lines). Right eye = OD; Left eye = OS

After nine months, BCVA in OD improved to 20/16 Snellen, but the patient still noticed a difference in quality of vision in OD, especially colors appeared less clear. BCVA in OS still remained unchanged. OCT imaging exhibited no detectable abnormalities in both eyes, signifying the complete restoration of retinal layers in OD (Fig. 7E2 and E4). AO-FIO imaging revealed cone mosaic patterns without discernible foveal lesions in both eyes compared to control 2 (Fig. 8). However, in both eyes the parafoveal cone density measurements was lower than the measurements of the age-matched controls (Fig. 9).

## Discussion and conclusion

The pathophysiology of ARPE remains elusive and diagnosing this disease remains challenging. The clinical features of ARPE are similar to those of several other diseases, including multiple evanescent white dot syndrome (MEWDS), central serous chorioretinopathy, pachychoroid disease, as well as the acute stage following light damage and acute macular neuroretinopathy [7]. A recent review questions the classification of ARPE as a distinct condition, noting that most cases reported in the literature are more accurately explained by other retinal diseases, such as MEWDS. Only 10% of cases could not be reclassified, indicating that ARPE is likely rare. The authors highlight the importance of multimodal imaging in refining diagnostic accuracy and establishing more precise criteria for diagnosing ARPE [8]. Other literature describes ARPE as a disease entity of its own and as part of a spectrum of self-limiting conditions affecting the RPE/photoreceptor interface, mostly occurring unilaterally [9]. Moreover, it stresses the importance of comprehensive multimodal imaging during the early stages and throughout the disease course for accurate diagnosis.

In the so far largest described case series (*N* = 18), OCT findings were analyzed in the acute phase of the disease. All patients showed an abnormal reflectivity in the RPE, 89% showed abnormal reflectivity in the EZ, and 7% in the ONL. This suggests that the origin of the disease takes place at the junction between RPE and cone photoreceptor outer segments [3]. Lu et al. described four patients, all displaying a dome-shaped hyper-reflective lesion at the ONL and disruption of the EZ and IZ. Within 3 months retinal recovery had been observed in a specific order: first external limiting membrane (ELM) recovery, followed by complete disappearance of the hyper-reflective lesions, and restoration of the EZ, and IZ. They suggest inflammation primarily affecting the outer neurosensory retina, with the IZ being the last structure to be restored [10]. 

Other case reports confirm these OCT findings of hyperreflectivity from the RPE layer to the ONL [11, 12]. They observe an accumulation of photoreceptor outer segment debris secondary to RPE dysfunction, which can occur after a viral infection [13]. The involvement of the EZ band and ONL could be associated with damage to the cone nuclei, which may lead to irreversible damage [14]. Few case reports describe ARPE post viral infection or post vaccination (Coxsackie virus A infection and corona virus disease-19 vaccination) [15–17]. 

In our study, both patients reported flu-like symptoms preceding the onset of ARPE. On OCT, we observed hyper-reflectivity in the fovea and parafoveal regions within the ONL, and an irregular pattern of the RPE in the fovea, with disruption of the EZ and IZ in all affected eyes. Notably, the ONL-RPE junction exhibited delayed recovery on OCT, consistent with prior research. AO-FIO imaging revealed decreased cone density compared to controls. The variability between the two controls is depicted in Figs. 4 and 8. Additionally the variability of cone density within control 1 at 2° temporal eccentricity was 26,132/mm^2^, 26,379/mm^2^, 26,624/mm^2^ respectively, measured over one year at three different time points. This indicates that the difference between patients and controls was substantially larger than the range of variability within our controls, potentially suggesting loss or damage of cone photoreceptors in ARPE patients. After more than six months, in AO-FIO the parafoveal cone density remained lower, and the lesions were still detectable. In contrast, in OCT complete recovery of all retinal layers could be observed. In addition, in patient 1, some hyporeflective spots in the foveal lesion on AO appear as black dots, resembling hyporeflective clumps. These hyporeflective clumps correspond to hyperreflective structures in the ONL on OCT and are suspect to be signs of inflammation [18–20]. 

The visibility of a cone photoreceptor depends on the preservation of its outer segment, which should remain unharmed and in contact with the apical processes of the RPE. In OCT, this appears as either an intact IZ or an EZ. In AO-FIO, the visibility of individual cones relies on the reflectance of the photoreceptors, with the IZ and EZ contributing substantially to this [21, 22]. The inability to visualize cones on AO-FIO imaging may suggest that the photoreceptors are damaged and potentially functioning less effectively. However, it is important to recognize that what we observe on AO-FIO may differ from the underlying histological processes. Furthermore the reflectivity of cones exhibits normal variability contingent upon the angle of illumination (Stiles Crawford effect) [23]. It is hypothesized that long-term variations in cone reflectivity are due to the renewal of the outer segments, while short-term variations are associated with nocturnal outer segment shedding [22, 24]. 

Nevertheless, it is essential to recognize that the visibility of cones also depends on the quality of the AO images, which can be influenced by instrument-related factors and/or ophthalmic factors in the anterior segment of the eye or the retina, such as cystoid macula edema [25]. To ensure the images are of the highest possible quality, a quality check was performed by the examiner during acquisition with the rtx1 camera. Additionally, the eyes examined were screened for ophthalmic factors that could affect image quality, but none were found. Furthermore, as the rtx1 camera cannot distinguish densely packed foveal cones, the central 4° area was excluded from the quantitative analysis to ensure it does not impact the findings.

Our AO observations support the hypothesis that the primary lesion of ARPE is situated at the junction EZ/IZ and the apical site of the RPE cells. Cone density measurements may be influenced by reduced reflectance of the IZ due to cellular inflammation. Although OCT images showed recovery of the IZ and EZ in both patients, the parafoveal cone density in AO-FIO did not return to the levels observed in the controls, indicating that possibly not all cones fully recovered. This observation may provide tentative support for the hypothesis that ARPE could potentially result in irreversible damage at the photoreceptor level. Although it did not affect the BCVA, according to the patients the quality of (color)vision had changed after the disease episode. To further examine this hypothesis, it may be advantageous to incorporate comparisons of AO-FIO with multifocal electroretinogram and microperimetry, thereby ensuring that cone function is adequately considered.

In conclusion, with AO-FIO imaging we have observed for the first time that ARPE may lead to irreversible cone photoreceptor cell loss, which could not be detected by OCT. This provides valuable insights into the pathology of ARPE and points out that AO-FIO could be a valuable additive imaging tool in diagnosing this disease and interpreting the long-term effects on the retinal cells.

## Data Availability

All data generated or analyzed during this study are available from the corresponding author on reasonable request.

## Abbreviations

ARPE: Acute retinal pigment epitheliitis
AO: Adaptive optics
AO-FIO: Adaptive optics flood illumination ophthalmoscopy
BCVA: Best corrected visual acuity
EZ: Ellipsoid zone band
ELM: External limiting membrane
FA-ICG: Fluorescent angiography-indocyanine green
FAF: Fundus autofluorescence
IZ: Interdigitation zone
OS: Left eye
OCT: Optical coherence tomography
ONL: Outer nuclear layer
RPE: Retinal pigment epithelium
OD: Right eye

## References

[1] Krill AE, Deutman AF. Acute retinal pigment epitheliitus. Am J Ophthalmol. 1972;74(2):193–205. 5054230 10.1016/0002-9394(72)90535-1

[2] Bowling B. Kanski’s Clinical Ophthalmology: A systematic approach. Elsevier; 2016.

[3] Cho HJ, Han SY, Cho SW, Lee DW, Lee TG, Kim CG, Kim JW. Acute retinal pigment epitheliitis: spectral-domain optical coherence tomography findings in 18 cases. Invest Ophthalmol Vis Sci. 2014;55(5):3314–9. 24787563 10.1167/iovs.14-14324

[4] Kim JE, Chung M. Adaptive optics for retinal imaging: current status. Retina. 2013;33(8):1483–6. 23538585 10.1097/IAE.0b013e31828cd053

[5] Roshandel D, Heath Jeffery RC, Charng J, Sampson DM, McLenachan S, Mackey DA, Chen FK. Short-term parafoveal cone loss despite preserved Ellipsoid Zone in Rod Cone Dystrophy. Transl Vis Sci Technol. 2021;10(14):11. 34904999 10.1167/tvst.10.14.11 PMC8684316

[6] Wooning SHP, Heutinck PAT, Liman K, Hennekam S, van Haute M, van den Broeck F, Leroy B, Sampson DM, Roshandel D, Chen FK, Pelt DM, van den Born LI, Verhoeven VMJ, Klaver CCW, Thiadens AHJ, Durand M, Chateau N, van Walsum T. Andrade De Jesus D and Sanchez Brea L. Automated Cone photoreceptors detection in adaptive Optics Flood-Illumination Ophthalmoscopy. Submitted to Ophthalmology Science; 2024. 10.1016/j.xops.2024.100675 PMC11925573 40114708

[7] Al-Nofal M, Charbel Issa P. Acute retinal pigment epitheliitis, a diagnostic myth? Eye (Lond). 2024;38(2):238–9. 37532834 10.1038/s41433-023-02683-w PMC10810810

[8] Fouad YA, Cicinelli MV, Marchese A, Casalino G, Jampol LM. Revisiting acute retinal pigment epitheliitis (krill disease). Surv Ophthalmol. 2024;69(6):916–23. 39025238 10.1016/j.survophthal.2024.07.003

[9] Casalino G, Viola F. Acute retinal pigment epitheliitis is not a diagnostic myth. Eye (Lond). 2023. 10.1038/s41433-023-02828-x PMC11009255 37968513

[10] Iu LPL, Lee R, Fan MCY, Lam WC, Chang RT, Wong IYH. Serial spectral-domain Optical Coherence Tomography findings in Acute Retinal Pigment Epitheliitis and the correlation to visual acuity. Ophthalmology. 2017;124(6):903–9. 28284786 10.1016/j.ophtha.2017.01.043

[11] González Escobar AB, Ibáñez García A, Chinchurreta Capote A, Gismero Moreno S, Lorenzo Soto M. Acute retinal pigment epitheliitis (ARPE). A case report. Arch Soc Esp Oftalmol (Engl Ed). 2022;97(4):230–3. 35523470 10.1016/j.oftale.2021.10.001

[12] Roy R, Saurabh K, Thomas NR. Multicolor Imaging in a case of Acute Retinal Pigment Epitheliitis. Retin Cases Brief Rep. 2021;15(1):45–8. 29474220 10.1097/ICB.0000000000000726

[13] Benlahbib M, Meziani L, Akesbi J, Nordmann JP. [Acute Retinal Pigment Epitheliitis: spectral-domain optical coherence tomography findings]. Épithélite rétinienne aiguë: apport de la tomographie par cohérence optique en Spectral Domain. J Fr Ophtalmol. 2015;38(4):333–9. 25838057 10.1016/j.jfo.2015.01.004

[14] Li YH, Chang YC, Wu WC. Spectral-domain optical coherence tomography finding of acute retinal pigment epitheliitis. Taiwan J Ophthalmol. 2019;9(4):276–9. 31942435 10.4103/tjo.tjo_23_19 PMC6947752

[15] Colucciello M. Acute Retinal Pigment Epitheliitis: Association with Acute Coxsackie a Virus infection. Retin Cases Brief Rep. 2023;17(5):504–6. 37643032 10.1097/ICB.0000000000001234

[16] Tajunisah I, Tan SS, Effendi-Tenang I, Samsudin A, Ling KP, Tan WY, et al. Case Report: Spectrum of interesting ocular manifestations following COVID-19 vaccination: a case series of real-world presentations. Front Cell Infect Microbiol. 2023;13:1243055. 37790912 10.3389/fcimb.2023.1243055 PMC10542575

[17] Muñoz-Solano J, Fernández-Avellaneda P, Gallego-Pinazo R, Dolz-Marco R. Atypical acute fovealitis in COVID-19 context. Am J Ophthalmol Case Rep. 2022;27:101641. 35782657 10.1016/j.ajoc.2022.101641 PMC9238021

[18] Amarasekera S, Williams AM, Freund KB, Rossi EA, Dansingani KK. Multimodal Imaging of Multifocal Choroiditis with adaptive Optics Ophthalmoscopy. Retin Cases Brief Rep. 2022;16(6):747–53. 36288621 10.1097/ICB.0000000000001134 PMC9606444

[19] Paques M, Meimon S, Rossant F, Rosenbaum D, Mrejen S, Sennlaub F, Grieve K. Adaptive optics ophthalmoscopy: application to age-related macular degeneration and vascular diseases. Prog Retin Eye Res. 2018;66:1–16. 30010022 10.1016/j.preteyeres.2018.07.001

[20] Pacini B, Bacherini D, Savastano A, Rizzo S, Caporossi T. Comparative analysis of macular microstructure in eyes treated with human amniotic membrane plug or internal limiting membrane transplant for failed Macular Hole. Acta Ophthalmol. 2022;100(4):e1031–5. 34562301 10.1111/aos.15013

[21] Jacob J, Paques M, Krivosic V, Dupas B, Couturier A, Kulcsar C, et al. Meaning of visualizing retinal cone mosaic on adaptive optics images. Am J Ophthalmol. 2015;159(1):118–23. e1. 25284764 10.1016/j.ajo.2014.09.043

[22] Litts KM, Cooper RF, Duncan JL, Carroll J. Photoreceptor-based biomarkers in AOSLO retinal imaging. Invest Ophthalmol Vis Sci. 2017;58(6):BIO255–67. 28873135 10.1167/iovs.17-21868 PMC5584616

[23] Nilagiri VK, Suheimat M, Lambert AJ, Turpin A, Vohnsen B, Atchison DA. Subjective measurement of the Stiles-Crawford effect with different field sizes. Biomed Opt Express. 2021;12(8):4969–81. 34513236 10.1364/BOE.427834 PMC8407820

[24] Kocaoglu OP, Liu Z, Zhang F, Kurokawa K, Jonnal RS, Miller DT. Photoreceptor disc shedding in the living human eye. Biomed Opt Express. 2016;7(11):4554–68. 27895995 10.1364/BOE.7.004554 PMC5119595

[25] Ashourizadeh H, Fakhri M, Hassanpour K, Masoudi A, Jalali S, Roshandel D, Chen FK. Pearls and pitfalls of adaptive Optics Ophthalmoscopy in inherited retinal diseases. Diagnostics (Basel). 2023;13(14). 10.3390/diagnostics13142413 PMC10377978 37510157

